# Supplementary material for: DMRforPairs: identifying Differentially Methylated Regions between unique samples using array based methylation profiles
Source: BMC Bioinformatics. 2014 May 15;15:141. doi: 10.1186/1471-2105-15-141 (PMC4046028; doi:10.1186/1471-2105-15-141)
Supplement: Additional file 2 — DMRforPairs output for the comparison of A431-MCF7 and NA17018-NA17105. Please start from the HTML files in each folder. Available via the BMC Bioinformatics website. [file 1471-2105-15-141-S2.zip › 1394847754114233_MOESM2_ESM/A431_MCF7/figures/25447.pdf]

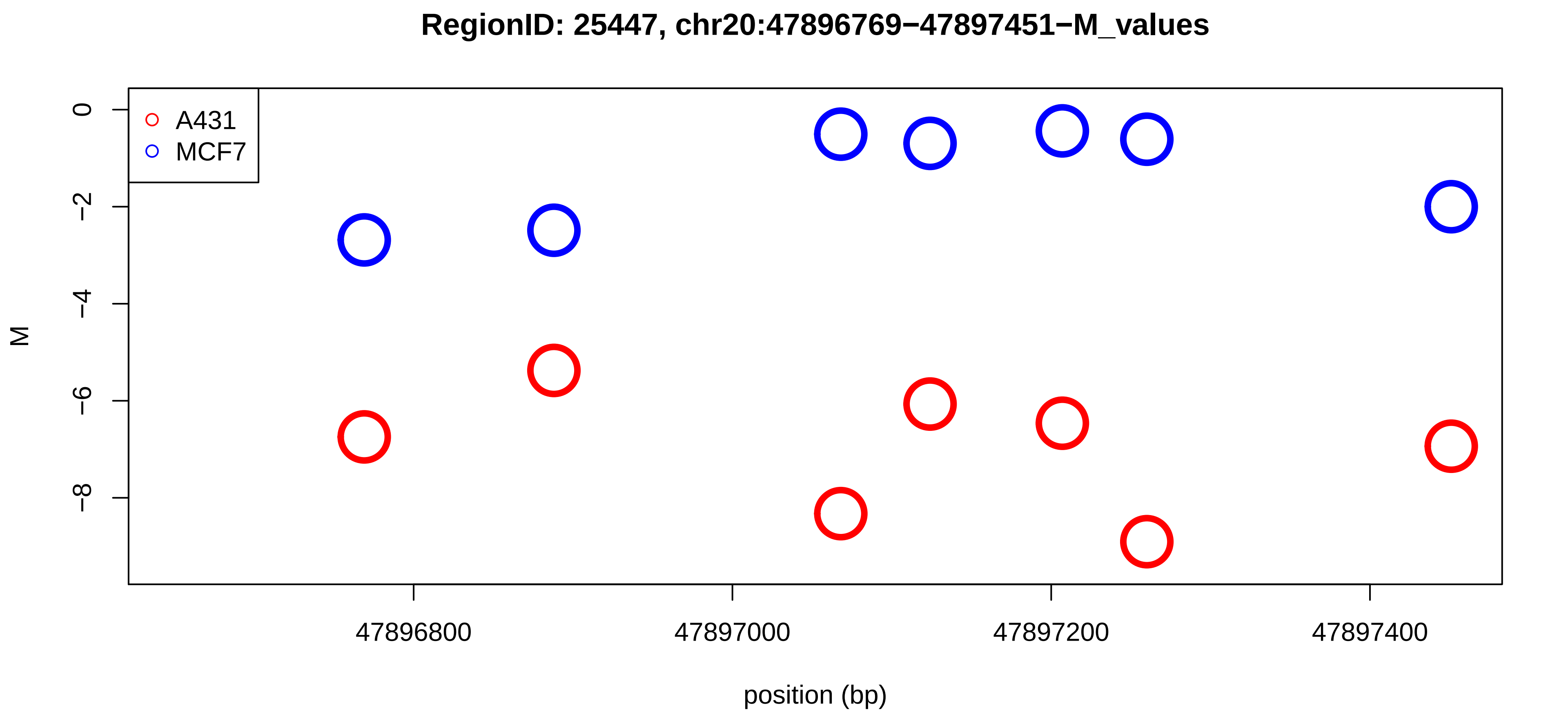
RegionID: 25447, chr20:47896769−47897451−M_values
A431
MCF7
0
−2
−4
−6
−8
M
47896800
47897000
47897200
47897400
position (bp)

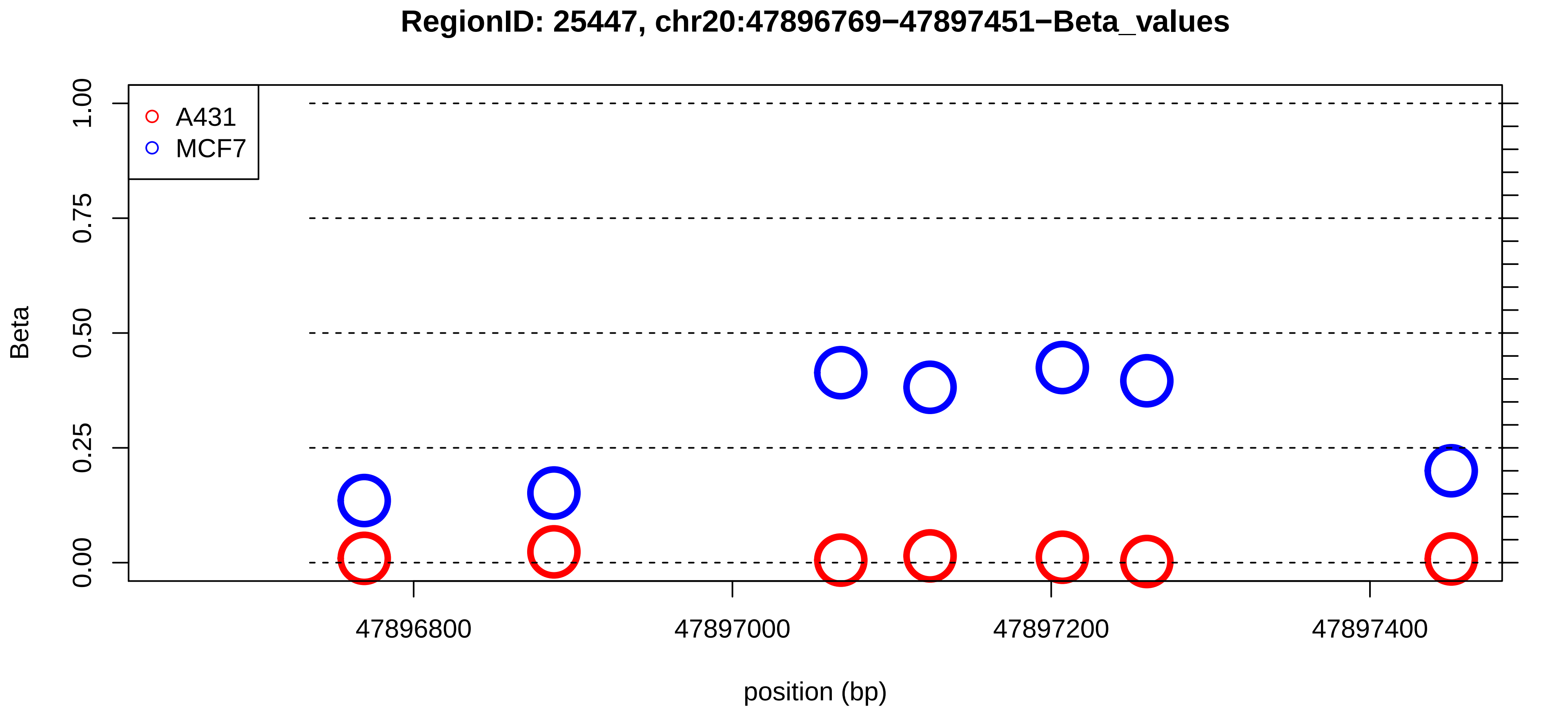
RegionID: 25447, chr20:47896769−47897451−Beta_values
A431
MCF7
1.00
0.75
0.50
0.25
0.00
Beta
47896800
47897000
47897200
47897400
position (bp)

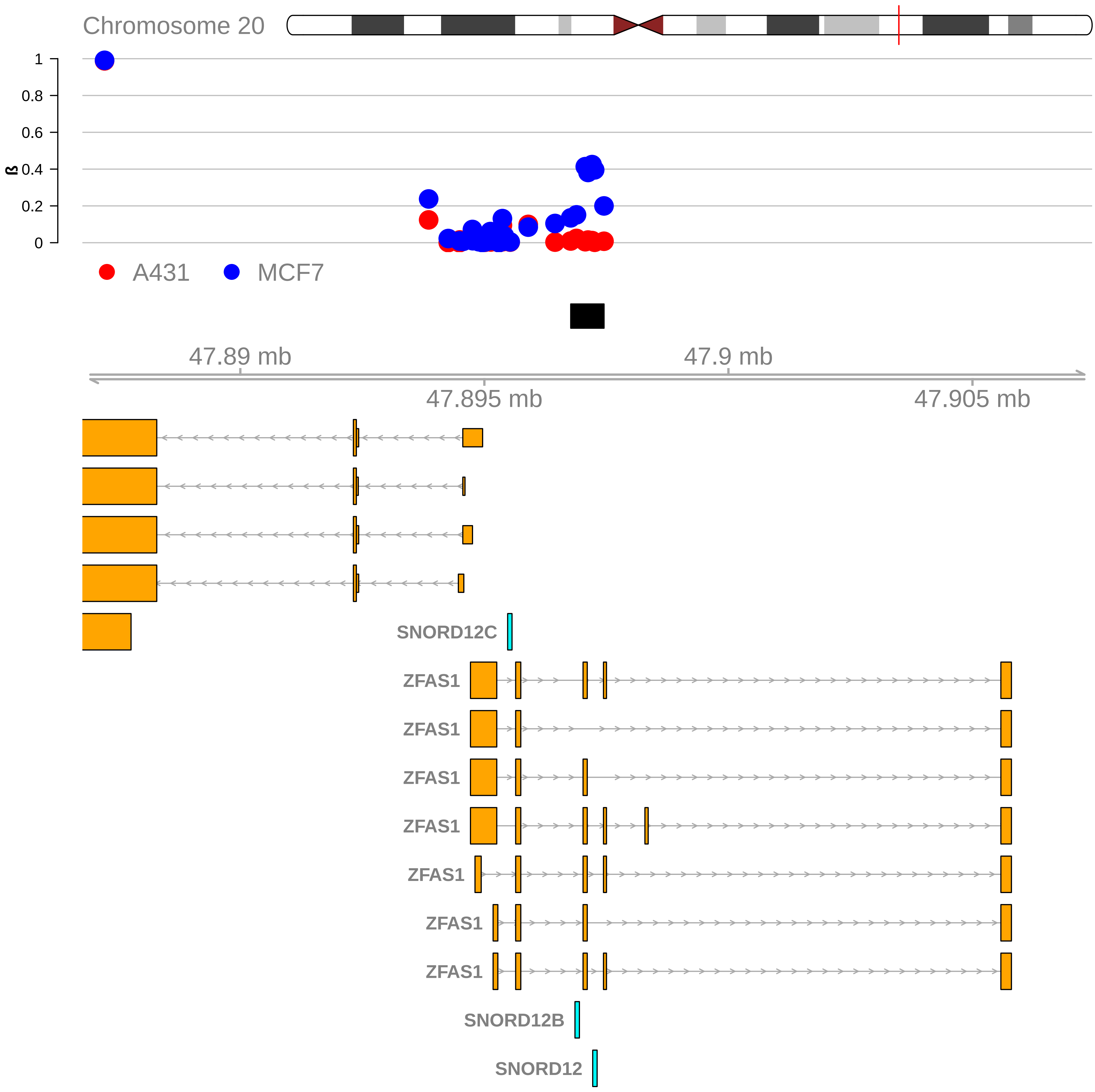

Chromosome 20
1
0.8
0.6
ß 0.4
0.2
0
A431
MCF7
47.89 mb
47.9 mb
47.895 mb
47.905 mb
SNORD12C
ZFAS1
ZFAS1
ZFAS1
ZFAS1
ZFAS1
ZFAS1
ZFAS1
SNORD12B
SNORD12
